# An Unusual Case of Evolving Localized Tetanus Despite Prior Immunization and Protective Antibody Titer

**DOI:** 10.7759/cureus.9498

**Published:** 2020-07-31

**Authors:** Biswaraj Tharu, Safa Ibrahim, Munir Shah, Sijan Basnet, Terrence Park

**Affiliations:** 1 Internal Medicine, Western Reserve Health Education/Northeast Ohio Medical University, Warren, USA; 2 Endocrinology and Diabetes, Baylor University Medical Center, Temple, USA; 3 Infectious Disease, Western Reserve Health Education/Northeast Ohio Medical University, Warren, USA; 4 Internal Medicine, Reading Hospital - Tower Health, Reading, USA

**Keywords:** evolving tetanus, localized tetanus, protective antibody titer, immunization, tetanus titer

## Abstract

Centers for Disease Control and Prevention has reported that tetanus infection in a fully immunized person with the last dose within 10 years is extremely rare. The prevalence of localized tetanus in such a scenario is unknown. Only two case reports of localized tetanus in previously immunized patients have been reported so far, making this the third one. Also, this is the first case of its kind to demonstrate evolving localized tetanus. Our patient is a 19-year-old man who presented with shortness of breath, pain in right upper extremity, shoulder, and neck. His chest X-ray and creatine kinase were normal. The patient was sent home. He presented again to our hospital two days later with difficulty swallowing and speaking as well as chest tightness. Routine blood tests, electrocardiogram, CT angiography of the chest, and transthoracic echocardiogram were normal. He gave a history of a cut in the right middle finger while removing the carpet a week before his presentation. His immunization history was complete with documented last tetanus shot nine years and two months ago. He was treated with tetanus vaccine and penicillin. His tetanus antitoxoid titer came out protective.

## Introduction

Tetanus vaccine has been very effective in the prevention of tetanus. Although the efficacy of tetanus toxoid has never been studied, it is inferred to be 100% because of the protective antitoxin levels after complete series [1]. Despite this, some patients mount low toxoid titers and are susceptible to tetanus. We present a case of a young male with localized tetanus that was evolving to generalized type despite previously immunization and good antibody titer.

## Case presentation

Our patient is a 19-year-old man with no significant past medical history presented to the emergency department (ED) with a sudden onset mild shortness of breath and sharp stabbing pain, 7/10 in the right upper extremity, shoulder, and neck. The patient denied fever, cough, runny nose, sore throat, ear pain, toothache, recent dental procedures, trismus, neck stiffness, chest pain, palpitation, nausea, vomiting, abdominal pain, hemoptysis, dysuria, hematuria, change in sensation/strength in the upper or lower extremities, back pain, peripheral edema, rash, ulcers, alcohol use, tobacco use, illicit drug use or, fall, depression/suicidal or homicidal ideation/hallucinations, change in body weight, bowel, or bladder habits. He did not have a past or family history of venous thromboembolism. On examination, his vitals were normal with an oxygen saturation of 97% in room air. The patient was anxious but did not have any significant physical exam findings. Creatine kinase, urine drug screen, chest x-ray, and urinalysis were normal. He was discharged home with a muscle relaxant.

Two days later, he again presented to ED with chest and back tightness leading to shortness of breath with associated difficulty in speaking, swallowing, and drinking and diffuse muscle pain and spasms. He stated that he was training for a half marathon and ran about every other day. A week ago, he had run 11 miles with no problem, and four days ago during a five-mile run, he had an episode where he was unable to breathe while running up a hill. However, he does not report fever, cardiac symptoms, or cardiac history in the family. His vitals were stable with an oxygen saturation of 99% on room air. The patient had stiffness of the right upper extremity. Although he complained of stiffness in the left arm, no stiffness was noted. Nuchal rigidity, Kernig's and Brudzinski's signs, and pharyngeal erythema were absent. Pertinent labs include white blood cell (WBC) count of 3.9 x 10^3^ cells/µL (reference ranges: 4-10.7 x 10^3^ cells/µL) and total creatine kinase of 101 units/L (reference ranges: 39-308 units/L) (Table 1). The electrocardiogram showed normal sinus rhythm with no acute ST-segment changes and serial troponins were negative. CT angiography of the chest was negative for pulmonary embolism. Transthoracic echocardiogram showed an ejection fraction of 60%, and stress testing was normal.

**Table 1:** Labs during first visit and two days after when he had second visit (day 1 and day 3) AST, aspartate transaminase; ALT, alanine aminotransferase

Labs	First visit	Second visit (day 1)	Second visit (day 3)	Reference range	Units
Hematology					
White blood cell count	5	3.9	4.3	4.3-10.7	×10^9^ cells/L
Red blood cell count	5.41	5.49	5.35	4.4-6.0	×10^6^ cells/L
Platelet count	197	191	148	135-435	×10^3^/µL
Hemoglobin	16.5	16.8	16.4	14-17	gm/dL
Chemistry					
Sodium	139	139	139	136-145	mmol/L
Potassium	3.9	3.4	3.6	3.5-5.1	mmol/L
Chloride	103	104	105	98-107	mmol/L
Anion gap	9	8	7	10.0-20.0	mmol/L
Blood urea nitrogen	16	19	13	7.0-18.0	mg/dL
Creatinine	1.12	1.4	1.1	0.70-1.25	mg/dL
Estimated creatinine clearance	102	63	108	107-139 (in males)	mL/min
Glucose	105	149	87	70-99	mg/dL
Lactic acid	2.1	N/A	N/A	0.5-2.2	mmol/L
Calcium	9.2	8.8	9	8.5-10.1	mg/dL
Total bilirubin	0.7	0.5	0.8	0.2-1.0	mg/dL
AST	28	24	32	10.0-37.0	units/L
ALT	37	35	40	12.0-78.0	units/L
Alkaline phosphatase	95	96	85	45-117	units/L
Total creatine kinase (CK)	174	101	82	39-308	units/L
CK-MB	1.3	<1	<1	0.5-3.6	ng/mL
CK-MB relative index	1	1	1	0-5	%
Troponin	0.043	0.019	0.017	0-0.045	ng/mL
Total protein	8.4	8.1	7.5	6.4-8.2	gm/dL
Albumin	4.4	4.3	4	3.4-5.0	gm/dL
Globulin	4	3.8	3.5	2.5-4.0	gm/dL
Lipase	165	N/A	N/A	73-393	units/L
Urine analysis (UA)	Negative	Negative	N/A		
Urine drug screen	Negative	Negative	N/A		
Diphtheria antitoxoid	N/A	N/A	1.36	>0.10	IU/mL
Tetanus antitoxoid	N/A	N/A	1.52	>0.10	IU/mL

Upon further review, the patient recalled cutting himself on the right middle finger with a nail while removing the carpet a week ago. This was followed by pain and stiffness in the right hand, but the patient did not seek medical care as it did not bother him. The patient and his mother reported completing all five childhood vaccinations with the last tetanus shot nine years and two months ago. No obvious injury was noted on his affected hand where he indicated he had a puncture wound. He had a carpopedal spasm of his right hand. Risus sardonicus, opisthotonus, Chvostek sign, and Trousseau’s sign were absent. His serum calcium was normal 9.2 mg/dL (ref: normal level 8.5-10.1 mg/dL). Tetanus antitoxoid titer and diphtheria antitoxoid titer were sent.

The patient was managed in the line of focal tetanus as per Infectious Disease service and started on penicillin G 3 million U every four hours. Since it was not a full blown tetanus and only had focal tetanus that was progressing to full tetanus, only tetanus vaccine was given, and human tetanus immune globulin (HTIG) was not given. He complained of spasms on his back and belly but objectively spasms were present on the right hands only. A dose of iv lorazepam 0.5 mg once was given. The next day he noted some improvement in symptoms. He received a tetanus vaccine and was discharged with a total of four days of penicillin treatment. After discharge, his tetanus antitoxoid titer (1.52 IU/mL; protective if ≥0.1 IU/mL) and diphtheria antitoxoid titer (1.36 IU/mL; protective if ≥0.1 IU/mL) came back protective.

## Discussion

Tetanus is one of the few bacterial diseases that do not confer immunity following recovery from an acute illness. Tetanus is acutely fatal and is caused by *Clostridium tetani* exotoxin tetanospasmin. *Clostridium tetani *is a gram-positive, anaerobic rod with drumstick appearance. The organism is sensitive to heat and oxygen contrary to the spore which is very resistant to phenol, common antiseptics, and autoclaving.

Clinically, there are three types of tetanus: generalized, cephalic, and local. Generalized tetanus is the most common form of tetanus (about 80% of reported tetanus). It has the classical triad of trismus, opisthotonus, and risus sardonicus. Cephalic tetanus is a rare form that presents with otitis media and involves cranial nerves, especially facial. Localized tetanus is uncommon and usually presents as persistent muscle contractions in the affected traumatic area. It mostly subsides in a week and can uncommonly transform into generalized tetanus; even so, the presentation would be milder form. Center for Disease Control and Prevention (CDC) has reported that it is extremely rare in an immunized person within the last 10 years to present with tetanus [1]. The prevalence of localized tetanus cases is such a scenario is unknown with only two reports so far [2,3]. Although CDC reports that in general very rarely can localized tetanus transform (about 1%) into fatal tetanus, there have not been any case reports/studies on localized tetanus, despite immunization transforming to generalized/fatal or even evolving types. This is the first case of its kind to report evolving localized tetanus.

The diagnosis of tetanus is entirely clinical. Diagnosis does not depend upon wound cultures as wound culture can be positive in people who do not have tetanus and only 30% of the cases have positive wound culture [1]. Patients with lower immunity or antitetanus antibodies have a high chance of tetanus infection. However, it is very important to note that the possibility of tetanus infection with protective levels of antibodies cannot be excluded [4]. 

Our case presentation indicates local tetanus evolving to regional. The patient had a history of cut injury in the right hand followed by pain and stiffness. These symptoms were tolerable enough for the patient not to seek medical care. His main complaint during the first ED visit was shortness of breath. During his second ED visit, he also had tightness/spasm in his trunk with difficulty in speaking, swallowing, and drinking and subjective bilateral upper extremities spasm (objectively only in the right extremity). The clinical presentation for those who have already received the tetanus vaccine seems to be less severe. This is also supported by Goulon et al. on 64 patients which showed that the severity of clinical presentation was inversely related to pretreatment antitetanus toxin antibody levels [5]. The development of localized tetanus in previously immunized could be because of low toxin load or can be an early manifestation of generalized tetanus [6].

This patient presented twice to the ED with nearly the same and evolving features. During the second admission, his symptoms got worse (subjective spasms and pain in bilateral upper extremities, more pronounced on the right) partly because the patient was already training for half-marathon (though creatine kinase levels for rhabdomyolysis was normal) and partly because the symptoms were worsening, shortness of breath and new tightness/spasm in axial structures (chest, shoulder, back, abdomen). Tetanus is a clinical diagnosis, and cultures and titers would not add any definitive conclusion to the management, so we proceeded with empirically treating the patient with penicillin G. The patient was given a tetanus vaccine despite his previous documented tetanus vaccine within last 10 years. When he showed signs of improvement with iv penicillin, he was discharged [7].

The differentials that can be mistaken for tetanus are meningitis, drug-induced dystonias, trismus due to dental infections, seizure, hypocalcemia, rabies, strychnine poisoning, stroke, malignant neuroleptic syndrome, and stiff person syndrome [7,8]. He did not have altered mentation, meningeal signs, or fever that rules out meningitis. Stroke was unlikely as he did not have any focal neurological deficit ruling out stroke. He did not have a toothache, recent dental procedures, or trismus that rules out dental infection. He did not have a history of animal bites to consider rabies. His labs did not show hypocalcemia. He did not have any aura, abnormal body movements, or postictal confusion seen with seizures. Stiff person syndrome can be ruled out as it is associated with type 1 diabetes mellitus, occurs in the second-fifth decade, presents with spasms of trunk and limbs, and is precipitated by auditory, tactile, or emotional stimuli, all of which were absent in our case. The fact that he did not have any history of drug use before presentation rules out drug-induced dystonia, malignant neuroleptic syndrome, and strychnine poisoning [7].

Although the efficacy of tetanus toxoid has never been studied, it is inferred to be 100% because of the protective antitoxin levels after complete series [1]. Therefore, it is a very effective vaccine. In the United States, protective immunity against tetanus is defined as an antitoxoid level of ≥0.1 IU/mL determined by ELISA (enzyme-linked immunosorbent assay). There are a lot of studies supporting the decennial tetanus booster. Hammarlund et al. in their cross-sectional analysis of 546 adults found that 99% of the adults had a protective level of antitoxoid titer 10 years after vaccination and the half-life of tetanus specific antibody was 14 years (95% CI, 11-17) [9]. Similarly, Borella-Venturini et al, in their 2017 study of 1,433 workers and students showed that 95% of the subjects had a protective antibody level of ≥0.1. Their study also suggested decennial booster is not needed for up to 20 years when the primary series of vaccination is complete [10]. Also in the correspondence published the same year, they emphasized that seroprotection to tetanus is not only based on the interval since the last dose but also the total number of vaccination received previously. For those who received five doses of immunization, the prevalence of non-seroprotected individuals was 3.1% contrary to 0% who received more than five scheduled doses [11]. All these studies point to the effectiveness of the vaccine. 

The development of vaccine failure or low levels of tetanus antitoxoid levels could be related to the patient factor per se (immune status, nutritional status), vaccine/logistics factor per se (cold chain storage), or improper interval dosing of the vaccine. The fact that our patient had a very good antibody titer suggests that the vaccine was of good quality and so was its cold chain of supply. 

## Conclusions

Immune response at around age 19 years unless otherwise should be very robust. With appropriate immunization and antibody titer, the chances of developing symptoms of tetanus should be very slim, if any. Our case has shown that tetanus can develop in an individual with good titer and can present with a milder form. One reason why this happens is because the protective cutoff value we refer to is inappropriately lower than what it actually should be, in which case the recommending committee should reconsider the guideline. The other reason could be that once in a while there may be outlier cases of disease despite immunization, the presentation of which is usually milder. This might lead to under-reporting of the disease especially the localized form of tetanus, by both patients and health care staff, delaying diagnosis and potentially increasing the risk until the disease evolves to severe form. The cause of development of tetanus even with a good anti-toxoid titer, without doubt, demands further scientific study and analysis. This also points to the need to formulate/update new insights and guidelines on surveillance such as disease preventable disease and clinical practice.
